# Role of S-Palmitoylation on IFITM5 for the Interaction with FKBP11 in Osteoblast Cells

**DOI:** 10.1371/journal.pone.0075831

**Published:** 2013-09-18

**Authors:** Takashi Tsukamoto, Xianglan Li, Hiromi Morita, Takashi Minowa, Tomoyasu Aizawa, Nobutaka Hanagata, Makoto Demura

**Affiliations:** 1 Graduate School of Life Science, Hokkaido University, Sapporo, Japan; 2 Faculty of Advanced Life Science, Hokkaido University, Sapporo, Japan; 3 Nanotechnology Innovation Station, National Institute for Materials Science, Tsukuba, Japan; University Paris Sud, France

## Abstract

Recently, one of the interferon-induced transmembrane (IFITM) family proteins, IFITM3, has become an important target for the activity against influenza A (H1N1) virus infection. In this protein, a post-translational modification by fatty acids covalently attached to cysteine, termed S-palmitoylation, plays a crucial role for the antiviral activity. IFITM3 possesses three cysteine residues for the S-palmitoylation in the first transmembrane (TM1) domain and in the cytoplasmic (CP) loop. Because these cysteines are well conserved in the mammalian IFITM family proteins, the S-palmitoylation on these cysteines is significant for their functions. IFITM5 is another IFITM family protein and interacts with the FK506-binding protein 11 (FKBP11) to form a higher-order complex in osteoblast cells, which induces the expression of immunologically relevant genes. In this study, we investigated the role played by S-palmitoylation of IFITM5 in its interaction with FKBP11 in the cells, because this interaction is a key process for the gene expression. Our investigations using an established reporter, 17-octadecynoic acid (17-ODYA), and an inhibitor for the S-palmitoylation, 2-bromopalmitic acid (2BP), revealed that IFITM5 was S-palmitoylated in addition to IFITM3. Specifically, we found that cysteine residues in the TM1 domain and in the CP loop were S-palmitoylated in IFITM5. Then, we revealed by immunoprecipitation and western blot analyses that the interaction of IFITM5 with FKBP11 was inhibited in the presence of 2BP. The mutant lacking the S-palmitoylation site in the TM1 domain lost the interaction with FKBP11. These results indicate that the S-palmitoylation on IFITM5 promotes the interaction with FKBP11. Finally, we investigated bone nodule formation in osteoblast cells in the presence of 2BP, because IFITM5 was originally identified as a bone formation factor. The experiment resulted in a morphological aberration of the bone nodule. This also indicated that the S-palmitoylation contributes to bone formation.

## Introduction

**Figure 1:**
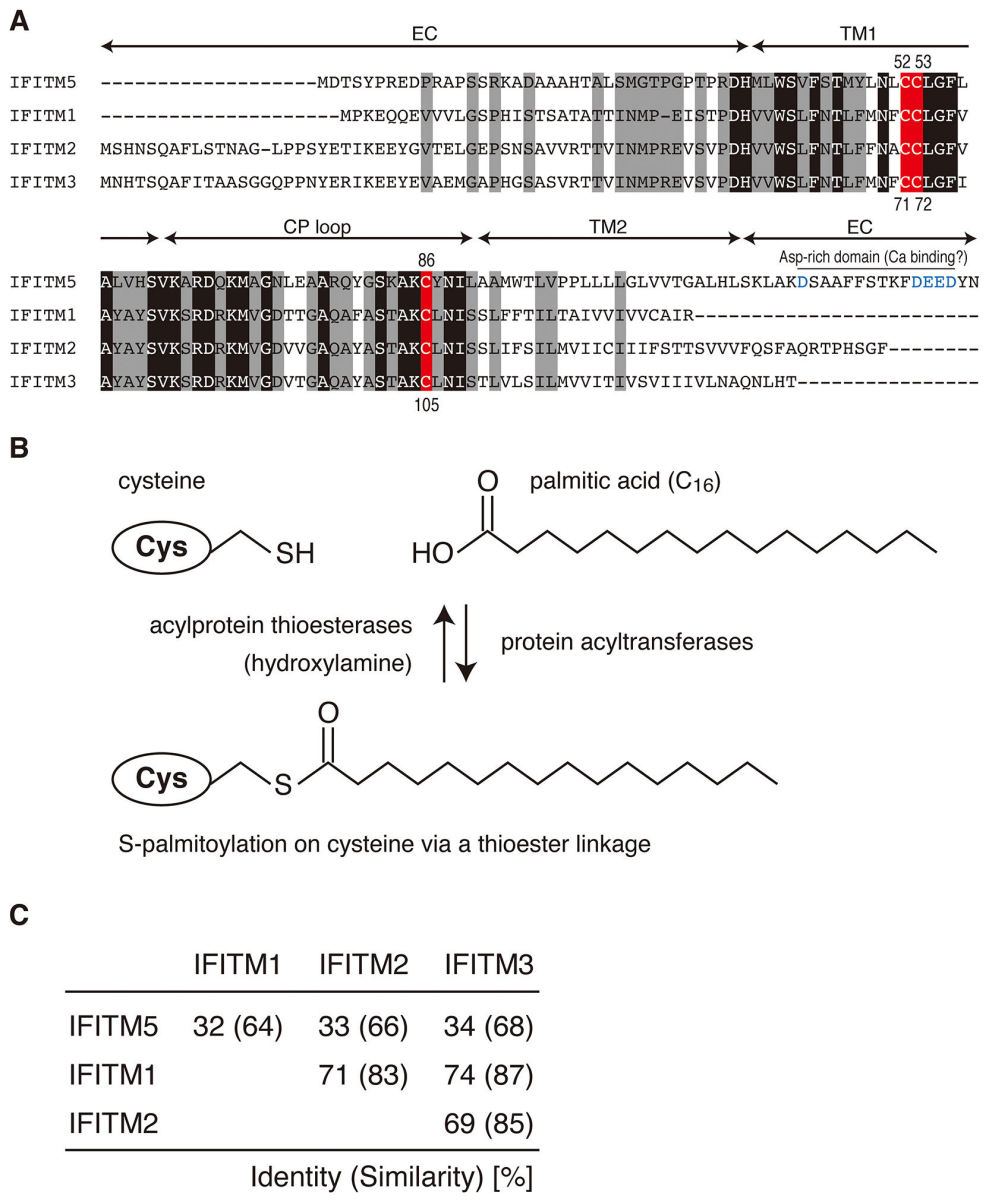
Comparison of the amino-acid sequences of IFITM proteins and illustration of protein S-palmitoylation. A) Amino-acid sequence alignment of IFITM5, IFITM1, IFITM2, and IFITM3 derived from mice. The conserved residues are highlighted in black. The three conserved cysteines are highlighted in red and numbered based on the sequence of IFITM5 (top) and IFITM3 (bottom). The residues unique in IFITM5 are highlighted in gray. The first and the second transmembrane domains, the extracellular sequences, and the cytoplasmic loop are indicated by arrows and denoted as TM1 and TM2, EC, and the CP loop, respectively. The TM domains were predicted by SOSUI. The aspartates at the C-terminal region in IFITM5 are shown in blue. B) The schematic illustration of the protein S-palmitoylation. The C_16_-palmitic acid is attached to cysteine via a thioester linkage. The palmitoylation and depalmitoylation are catalyzed by protein acyltransferases and acylprotein thioesterases, respectively. In this study, hydroxylamine, NH _2_OH, was used to reduce the thioester linkage. C) The amino acid sequence identity (similarity) among IFITM5, IFITM1, IFITM2, and IFITM3 is summarized.

The interferon-induced transmembrane (IFITM) protein family (also known as the Fragilis family in mice) is a part of the dispanin family [1] and is composed of double-transmembrane α-helices connected by a cytoplasmic (CP) loop and extracellular (EC) amino- and carboxyl-terminal polypeptide sequences (Figure 1-A). The IFITM proteins are evolutionarily conserved in vertebrates [2]. Recent genomic research has revealed that there are 5 IFITM members in humans (IFITM1, 2, 3, 5 and 10) and 7 members in mice (IFITM1, 2, 3, 5, 6, 7, and 10). These proteins play roles in diverse biological processes, such as germ cell maturation during gastrulation (IFITM1-3) [3-5], cell-to-cell adhesion (IFITM1) [6-8], antiviral activity (IFITM1-3) [9-17], and bone formation (IFITM5) [18-22], although the detailed functions of IFITM6, 7, and 10 are unknown at present. In particular, IFITM3 has been a target of intensive studies on its activity against influenza A (H1N1) virus infection and internalization [9-14].

In 2010, Dr. Yount and co-workers reported that the antiviral activity of IFITM3 is dependent on S-palmitoylation on the protein [10]. The S-palmitoylation [23] is a post-translational modification on proteins by C_16_ saturated-fatty acids (palmitic acids) covalently attached to certain cysteine residues via a thioester linkage (Figure 1-B). The modification is reversibly catalyzed by protein acyltransferases and acylprotein thioesterases, and confers unique properties to the protein, such as membrane binding and targeting, immunoreactivity, and protein-protein interaction. The authors revealed that IFITM3 is S-palmitoylated on three membrane proximal cysteines, Cys71 and Cys72 in the first transmembrane (TM1) domain, and Cys105 in the CP loop (Figure 1-A) [10]. In addition, IFITM3 lacking the S-palmitoylation is not clustered in the cell membrane and significantly diminishes the antiviral activity. Moreover, the cysteines in IFITM2, Cys70, Cys71, and Cys104 are also palmitoylated in the same manner, which affects the intracellular localization [24]. A resent study has revealed that murine IFITM1 has four cysteine residues (Cys49, Cys50, Cys83, and Cys103) for the S-palmitoylation, which is required for the antiviral activity and the protein stability [25]. The other IFITM family members also possess these cysteines (Figure 1-A), and thus the role of the S-palmitoylation on the cysteines should be significant for the functions of IFITM proteins.

Here, we focused on IFITM5, which is also known as bone-restricted IFITM-like (BRIL) protein [18]. Among the IFITM family proteins, IFITM5 is unique. (i) *Expression of IFITM5*: Unlike the other IFITM family proteins, the expression of IFITM5 is not induced by interferons because the region upstream of the *ifitm5* gene lacks the interferon regulatory elements [26]. Furthermore, the expression of IFITM5 is mostly restricted to osteoblast cells [18,19,27], while the other IFITM proteins are expressed ubiquitously (*ii*). *Amino-acid sequence similarity*: The amino acid sequence of IFITM5 is relatively dissimilar to IFITM1-3 proteins (~ 65% similarity), while IFITM1-3 proteins share ~ 85% similarity with each other (Figure 1-C). In addition, IFITM5 has an aspartate-rich domain in the C-terminal region, which could be involved in calcium binding (Figure 1-A) [26]. (iii) *Role of IFITM5 in bone formation*: The expression of IFITM5 is associated with mineralization during the bone formation process in osteoblast cells [18-21]. Previous studies have confirmed the expression of IFITM5 in bone tissues in mice, rats, humans and tammar wallabies [2]. The *ifitm5*-gene knockout mice have smaller bones [19]. Moreover, the knockdown of the *ifitm5* gene by small hairpin RNA induces a decrease in bone nodule formation, whereas overexpression of the gene in UMR106 cells has been shown to increase calcium uptake and bone nodule formation [18]. (iv) *Role of IFITM5 for immune activity*: Recent studies have revealed that IFITM5 interacts with the FK506-binding protein 11 (FKBP11) to form IFITM5-FKBP11-CD81-the prostaglandin F2 receptor negative regulator (FPRP) complex [28]. When the complex is formed, the expressions of 5 interferon-induced genes are induced, including bone marrow stromal cell antigen 2 (*Bst2*), interferon inducible protein 1 (*Irgm*), interferon-induced protein with tetratricopeptide repeats 3 (*Ifit3*), b(2)-microglobulin (*B2m*), and MHC class I antigen gene. Consequently, these results indicate that IFITM5 is involved not only in the bone formation but also in the immune system activity.

In this study, we investigated the S-palmitoylation of IFITM5 and its role in the interaction with FKBP11 in mouse osteoblast cells. Cells transfected by a plasmid DNA encoding mouse IFITM5 were grown in the presence of an established chemical reporter, 17-octadecynoic acid (17-ODYA) [29,30], or an inhibitor for the S-palmitoylation, 2-bromopalmitic acid (2BP) [31]. The biochemical assays using these compounds revealed that the wild-type IFITM5 is S-palmitoylated. To identify the S-palmitoylation site in IFITM5, we prepared cysteine-substituted mutants, IFITM5-C86A, -C52A/C53A, and -C52A/53A/86A (Cys-less). The chemical reporter assay suggested that at least two out of three cysteines in IFITM5 are S-palmitoylated. The interaction of IFITM5 with FKBP11 was examined by immunoprecipitation assay, resulting in the loss of the interaction in the presence of 2BP. The same result was obtained in the two mutants, C52A/C53A and Cys-less. These results suggested that the S-palmitoylation on Cys52 and/or Cys53 in the TM1 domain of IFITM5 is necessary for the interaction with FKBP11. On the other hand, Cys86 in the CP loop of IFITM5 was S-palmitoylated but not involved in the interaction. Because this interaction is important for the immunologically relevant gene expression, it was indicated that the role of the S-palmitoylation is to promote the interaction of IFITM5 with FKBP11 and to regulate the immune activity in the osteoblast cells. The possible interaction mechanism and the effect of the S-palmitoylation on the bone nodule formation will be discussed.

## Materials and Methods

### 1. Recombinant DNA construction

For mammalian cell expression, plasmid vectors of wild-type IFITM5 (IFITM5-WT) and FLAG-fused FKBP11 (FKBP11-FLAG) were constructed by inserting the cloned genes into a pBApo-CMV Neo expression vector (Takara Bio, Shiga, Japan). The details of the recombinant DNA constructs were the same as described previously [19]. The genes of IFITM5 mutants (IFITM5-C86A, -C52A/53A, and -C52A/C53A/C86A (Cys-less)) were prepared using a QuikChange site-directed mutagenesis kit (Stratagene, La Jolla, CA). The plasmid vectors of FLAG-fused IFITM5-WT, -C52A/53A, and Cys-less were constructed by inserting the cloned genes into the pBApo-CMV Neo expression vector. For *E. coli* cell expression, the plasmid vector of IFITM5-WT was constructed by inserting the cloned gene into a pET22b (Novagen, Madison, WI) expression vector. The forward primer 5’-GGAATTCCATATGGACACTTCATATCCCCGTG-3’ and the reverse primer 5’-CCGCTCGAGGTTATAGTCCTCCTCATCAAACTTGG-3’ were used to amplify the gene encoding the entire IFITM5 from the plasmid vector for mammalian cell expression described above. The underlined letters denote an NdeI and an XhoI cleavage site, respectively. The plasmids of IFITM5 mutants were prepared using a QuikChange site-directed mutagenesis kit. The sense and anti-sense primers used were 5’-GGCAGTATGGCTCCAAAGCCAAGGCGTACAACATCCTGGCTGC-3’ and 5’-GCAGCCAGGATGTTGTACGCCTTGGCTTTGGAGCCATACTGCC-3’ for IFITM5-C86A; and 5’-GCACGATGTACCTGAATCTGGCGGCGCTTGGATTCCTGGCGC-3’ and 5’-GCGCCAGGAATCCAAGCGCCGCCAGATTCAGGTACATCGTGC-3’ for IFITM5-C52A/C53A, respectively (Sigma-Aldrich, St. Louis, MO).

### 2. Mammalian cell culture, transfection, and metabolic labeling

Osteoblast-like MC3T3 cells were provided by the RIKEN, Cell Bank (RCB 1126). The procedures for cell culture, transfection, and protein expression were the same as reported previously. When necessary, 2-bromopalmitic acid (2BP; Wako, Osaka, Japan) and 17-octadecynoic acid (17-ODYA; Sigma-Aldrich) were dissolved in 99.5% dimethyl sulfoxide (DMSO; Wako) and added to differentiation medium at concentrations of 100 μM and 50 μM in less than 0.1% DMSO, respectively [30,31].

### 3. E. coli cell culture, protein expression, and purification

Wild-type and mutant IFITM5 proteins were also produced using an *E. coli* recombinant expression system. *E. coli* BL21(DE3) cells transformed by the expression plasmid were grown at 37°C in LB medium containing 50 μg/mL ampicillin. After four-hour induction by 1 mM isopropyl β-D-thiogalactopyranoside (IPTG), cells were harvested by centrifugation (6,400 × g for 10 min at 4°C). The cells were suspended in 50 mM Tris-HCl buffer (pH 8) and disrupted by a French press (Ohtake, Tokyo, Japan) (100 MPa × 4 times). The crude membrane fraction was collected by ultracentrifugation (178,000 × g for 90 min at 4°C). The collected fraction was solubilized with 1.5% n-dodecyl-β-D-maltopyranoside (DDM) (Dojindo Lab, Kumamoto, Japan) in 50 mM Tris-HCl, pH 8, containing 0.3 M NaCl and 5 mM imidazole. After the ultracentrifugation, the supernatant was incubated with Ni^2+^-NTA agarose resin (Qiagen, Hilden, Germany). The resin was applied to a chromatography column and washed with 50 mM imidazole containing 50 mM Tris-HCl (pH 8), 0.3 M NaCl and 0.1% DDM. The DDM-solubilized IFITM5 was collected by elution with the same buffer containing 0.3 M imidazole. The sample media were replaced by the appropriate buffer solution by two passages over a PD-10 column (GE Healthcare UK, Ltd., Amersham Place, England).

### 4. Immunoprecipitation and western blot

The experimental details are described in previous reports [19,28]. Briefly, total proteins were extracted from the osteoblast cells which co-expressed IFITM5 and FKBP11-FLAG using a total protein extraction kit (BioChain Institute Inc., Newark, CA). Then, the cell lysate was incubated with anti-FLAG M2 agarose gel (Sigma-Aldrich) at 4°C for 2 h. To recover FKBP11-FLAG, 500 ng/μL 3 × FLAG peptide (Sigma-Aldrich) dissolved in Tris-buffered saline was added to the collected gel at 4°C for 1 h. The recovered proteins and the cell lysate containing total proteins were analyzed by SDS-PAGE (15% ePAGEL; ATTO, Tokyo, Japan) and western blot. The anti-IFITM5 polyclonal antibody, which was prepared from the amino-terminal peptide sequence (TSYPREDPRAPSSRC), and anti-FLAG monoclonal antibody (Sigma-Aldrich) were used as primary antibodies. The HRP-conjugated goat anti-rabbit IgG (H+L) (Zymed Laboratories, San Francisco, CA) and goat anti-mouse IgG (H+L) (Sigma-Aldrich) antibodies were used as secondary antibodies for the anti-IFITM5 and anti-FLAG primary antibodies, respectively. The proteins were detected by chemiluminescent reaction (MercK-Millipore, Billerica, MA).

### 5. Fluorescent labeling of 17-ODYA-labeled IFITM5 proteins

The cell lysate extracted from the osteoblast cells metabolically labeled by 17-ODYA was incubated with anti-FLAG M2 agarose gel to obtain purified FLAG-fused IFITM5 proteins. The 17-ODYA-labeled proteins were chemically labeled with azide-PEG_3_-5(6)-carboxytetramethylrhodamine (TAMRA-azide; Click Chemistry Tools, Scottsdale, AZ) with reference to previous studies [10,29,30,32] and the manufacturer’s guide. The proteins separated by SDS-PAGE were visualized using a 532-nm laser for excitation and the fluorescence by TAMRA (565 nm) was detected using a 575-nm long-path filter (Typhoon FLA 9000; GE Healthcare).

### 6. Examination of bone nodule formation

The subcultured osteoblast MC3T3 cells were seeded at a density of 5,000 cells/cm^2^ in 40 mm dishes and cultured in α-Modified Eagle’s Medium (α-MEM; Sigma-Aldrich) containing 10% (v/v) fetal bovine serum (FBS; Nichirei Biosciences Inc., Tokyo, Japan). On the next day, this was replaced with differentiation medium, containing 2 mM glycerophosphate and 50 μg/mL sodium ascorbate at final concentrations, to induce osteoblast differentiation. When necessary, 100 μM 2BP in less than 0.1% DMSO, or 0.1% DMSO alone was added to the differentiation medium at final concentrations. All cultures were incubated at 37°C in a humidified atmosphere containing 5% CO_2_ for 27 days. Mineralized nodules were stained with Alizarin Red S (Sigma-Aldrich). The standard staining procedure was used. The mineralized nodules were checked every three days.

## Results

### 1. Identification of S-palmitoylation on IFITM5

**Figure 2:**
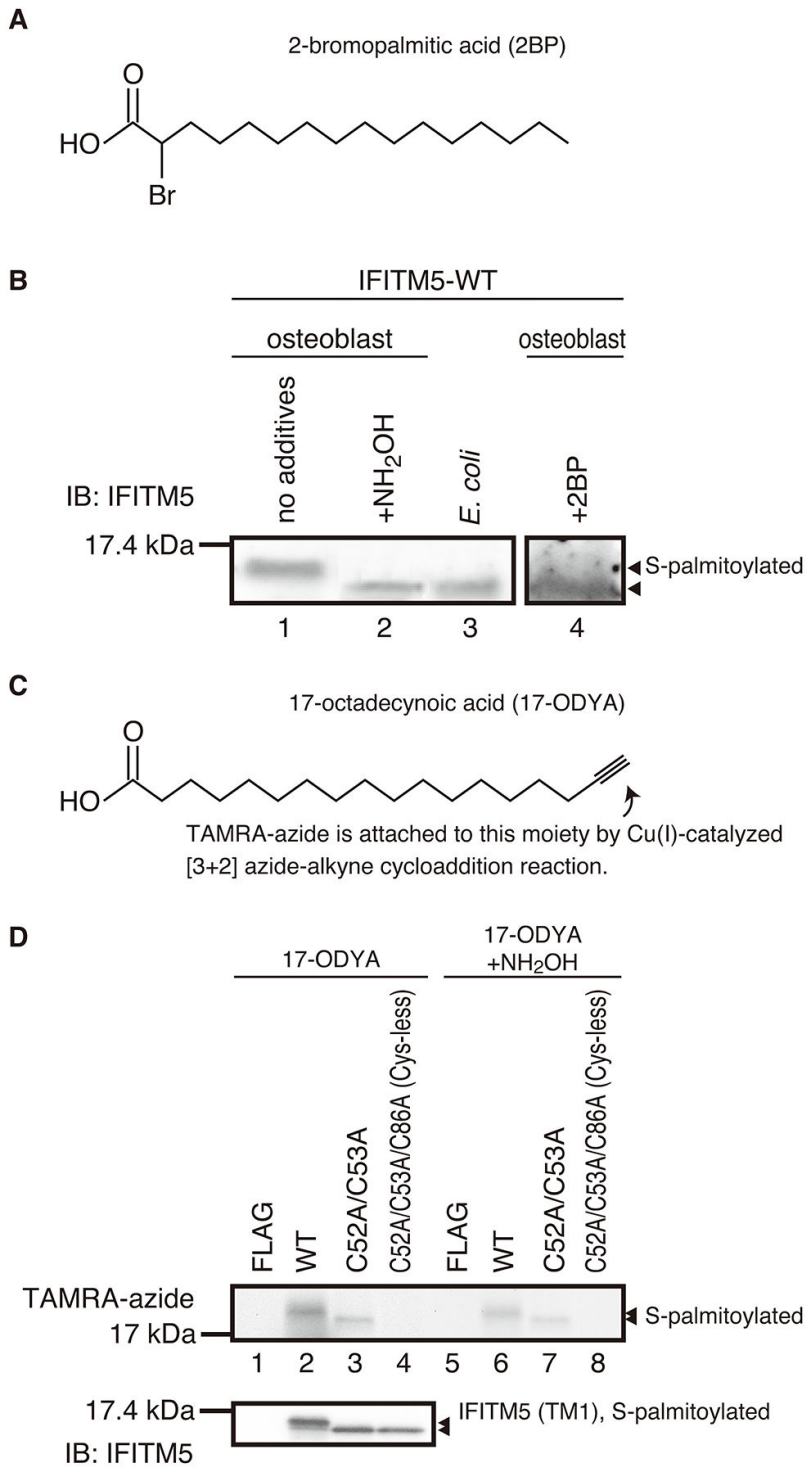
Biochemical analyses for the S-palmitoylation on IFITM5. A) Molecular structure of 2-bromopalmitic acid (2BP). B) Western blot for the IFITM5-WT expressed in the osteoblast cells in the presence and the absence of 2BP (lanes 1 and 4), and IFITM5-WT expressed in the *E. coli* cells (lane 3). Note that the lane 4 is repeated from the lane 2 of Figure 4-A. The same sample used in lane 1 was treated with 2.5% (w/v) neutral hydroxylamine (NH_2_OH) and applied in lane 2. Arrows indicate the high molecular-mass form (upper) and low molecular-mass form (lower), corresponding to the S-palmitoylated and depalmitoylated forms of IFITM5-WT, respectively. The experiment was carried out 3 times. C) Molecular structure of 17-octadecynoic acid (17-ODYA). D) In-gel fluorescent image of 17-ODYA/TAMRA-labeled IFITM5 proteins before (left panel) and after (right panel) the treatment with 2.5% (w/v) neutral hydroxylamine (NH_2_OH). Arrows indicate the S-palmitoylation on IFITM5 proteins. The lower panel shows the results of the western blot for the 17-ODYA-labeled IFITM5 proteins. The fully labeled form (WT, lane 2) and partially (C52A/C53A, lane 3) and completely (Cys-less, lane 4) labeled forms are seen. The experiment was repeated 2 times.

To identify the S-palmitoylation on IFITM5, the osteoblast cells harboring the plasmid DNA encoding IFITM5-WT were cultured in the absence and presence of 2BP, which inhibits the S-palmitoylation (Figure 2-A) [31]. Then, the cell lysate containing total protein was extracted for use in the SDS-PAGE and western blot analyses. For purposes of comparison, *E. coli* cells were also cultured in the absence of 2BP and the cell lysate was extracted. Figure 2-B shows the results of the western blot assay for IFITM5-WT expressed in the osteoblast and the *E. coli* cells. In the osteoblast cells, IFITM5-WT exhibited a single band near the 17.4 kDa molecular-mass marker (see lane 1) in the absence of 2BP. However, in the presence of 2BP (see lane 4), the band appeared at a lower position than that in the absence of 2BP (lane 1). These results suggested that IFITM5-WT has high and low molecular-mass forms in the absence and presence of 2BP, respectively. The S-palmitoylation is a reversible reaction, and therefore is depalmitoylated by a strong reductant such as hydroxylamine [10]. Following hydroxylamine treatment (see lane 2), the band appeared at the same position as in the presence of 2BP (lane 4). In prokaryote *E. coli* cells, the post-translational modification does not occur. Hence, the band was also observed at the same lower position (see lane 3). In the case of IFITM3, the palmitoylation was also reported to induce a change in mobility on electrophoresis, just as in our present results [10].

For direct observation of the S-palmitoylation, an established chemical reporter, 17-ODYA (Figure 2-C), was used. The osteoblast cells harboring the plasmid encoding IFITM5-WT were cultured in the presence of 17-ODYA to label the protein metabolically. Following the extraction and the purification of the cell lysate, the labeled IFITM5-WT was ligated with TAMRA-azide according to the Cu(I)-catalyzed [3+2] azide-alkyne cycloaddition method [10,29,30,32]. An in-gel fluorescence image of the 17-ODYA-TAMRA-labeled IFITM5-WT (see lane 2 in Figure 2-D) showed that IFITM5 was S-palmitoylated in the osteoblast cells. The FLAG-tag attached to IFITM5 has no influence on the modification and chemical labeling (lanes 1 and 5). In addition, after the hydroxylamine treatment (see lane 6), the fluorescence became weak because of the dissociation of 17-ODYA from IFITM5, which was the same mechanism as the dissociation of the palmitic acid from IFITM5 by reduction as described above (lane 2 of Figure 2-B).

Therefore, we concluded that the IFITM5 expressed in the native osteoblast cells is S-palmitoylated. In addition, the bands corresponding to the high and the low molecular-mass forms shown in western blot analysis were tentatively assigned to the S-palmitoylated and the depalmitoylated forms, respectively.

### 2. S-palmitoylation site of IFITM5

**Figure 3:**
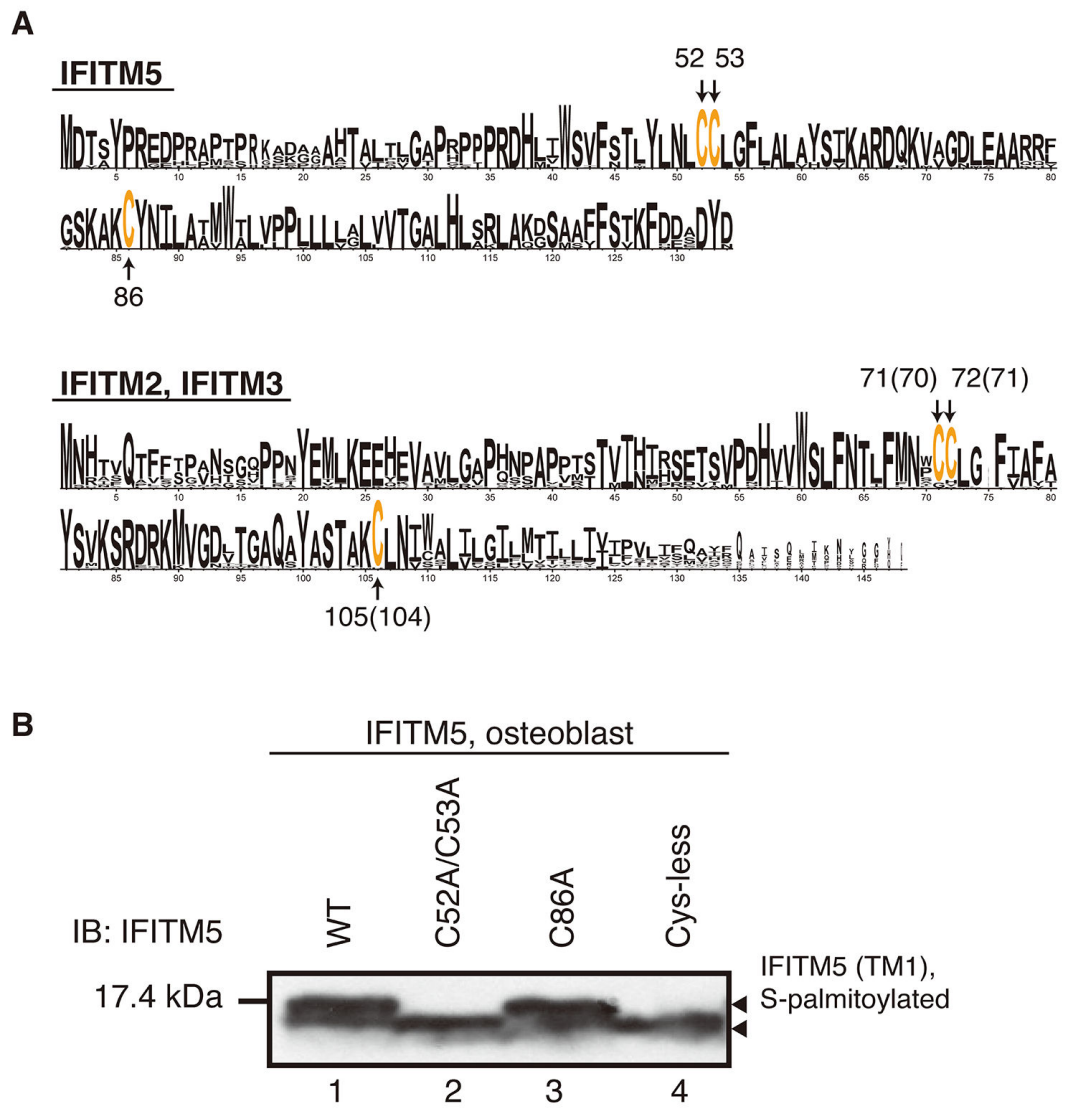
S-palmitoylation sites on IFITM5. A) Amino acid occurrence in putative mammalian homologs of IFITM5 (upper panel), and IFITM2 and IFITM3 (lower panel). The conserved cysteines are highlighted in orange and numbered. In the lower panel, the numbers given in parenthesis correspond to the residual number for IFITM2. For the calculation of probability, a total of 23 IFITM2, 23 IFITM3, and 17 IFITM5 sequences derived from mammalian species in the Kyoto Encyclopedia of Genes and Genomes (KEGG) database were used. Sequence alignment was carried out using CLUSTALW. Sequence logos were generated using WEBLOGO 3. B) Western blot for the wild-type and cysteine-substituted mutants of IFITM5 expressed in the osteoblast cells. For detection, the anti-IFITM5 antibody was used as a primary antibody. The upper arrow indicates that C52 and/or C53 in the TM1 domain is S-palmitoylated (lanes 1 and 3). The C52A/C53A (lane 2) and Cys-less (lane 4) mutants are partially and completely depalmitoylated. The experiment was carried out 2 times.

As described above in the *Introduction*, cysteine residues are the substrate for S-palmitoylation. IFITM5 possesses three cysteines, Cys52 and Cys53 in the TM1 domain, and Cys86 in the CP loop (Figure 1-A). All of these cysteines are highly conserved among the mammalian IFITM family proteins (Figure 3-A). To identify the modification site in IFITM5, we prepared cysteine-substituted mutants, IFITM5-C52A/C53A, -C86A, and -C52A/C53A/C86A (Cys-less). The osteoblast cells harboring each plasmid were cultured in the absence of 2BP, and then the cell lysate was extracted. Figure 3-B shows the results of the western blot detecting the expression of all the mutants in the osteoblast cells. In the C52A/C53A and Cys-less mutants (see lanes 2 and 4), the low molecular-mass form was detected. This result indicates that either Cys52 or Cys53 is involved in the S-palmitoylation. In addition, as shown in Figure 2-D, strong and weak fluorescence were detected in the C52A/C53A mutant in the absence and presence of hydroxylamine (lanes 3 and 7), respectively, but not in the Cys-less mutant (lanes 4 and 8). These results suggested that the rest of the cysteine in the C52A/C53A mutant, Cys86, is S-palmitoylated and the Cys-less mutant completely lost the S-palmitoylation because all the cysteines were substituted. Therefore, we concluded that Cys86, plus one or two other cysteine residues in IFITM5, i.e., Cys52 and/or Cys53, are S-palmitoylated. In addition, it was found that the S-palmitoylation on the TM1 domain has a major effect on the mobility in the gel (lower panel of Figure 2-D and Figure 3-B). Therefore, we hereafter refer to the high and low molecular-mass forms as the TM1-palmitoylated and the TM1-depalmitoylated forms, respectively. Finally, we reassigned the bands shown in the western blot analysis as follows: IFITM5-WT is fully palmitoylated, the C86A mutant is partially palmitoylated at Cys52 and/or Cys53, the C52A/C53A mutant is partially palmitoylated at Cys86, and the Cys-less mutant is completely depalmitoylated.

### 3. Role of S-palmitoylation for the IFITM5-FKBP11 interaction

**Figure 4:**
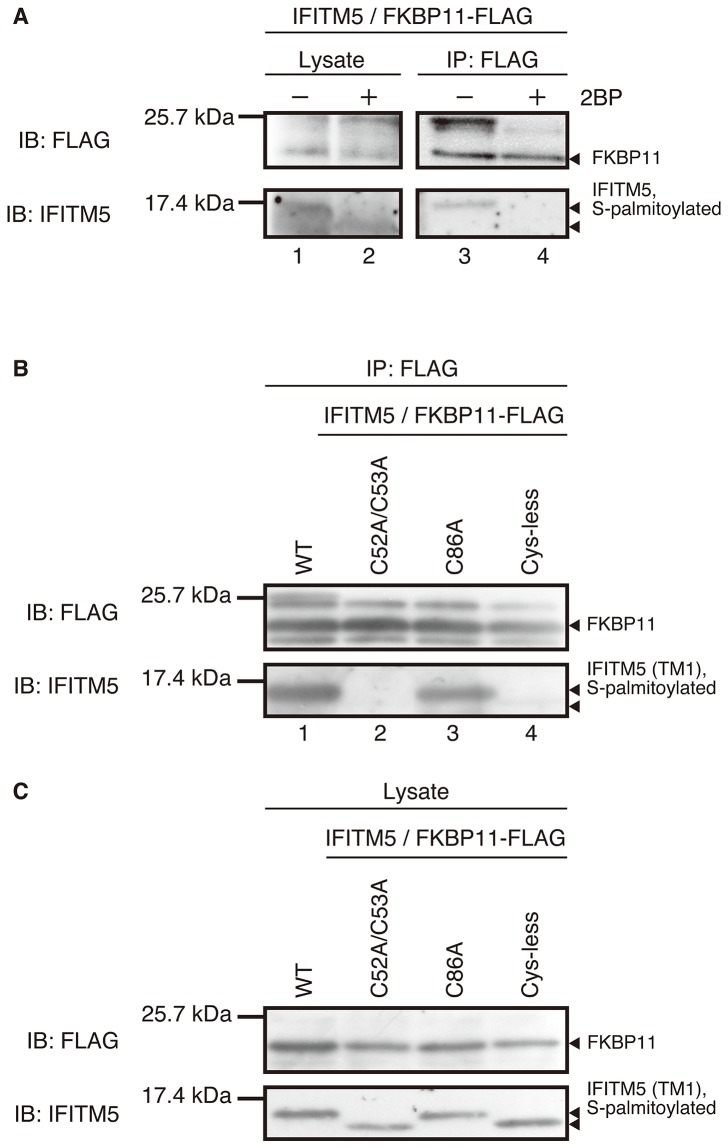
Western blot for detecting the interaction of IFITM5 with FKBP11. For detection of FKBP11-FLAG (upper panels) and IFITM5 (lower panels), the anti-FLAG and the anti-IFITM5 antibodies were used as primary antibodies, respectively. Arrows indicate the existence of each protein and the S-palmitoylation on IFITM5. A) Western blot for the co-immunoprecipitation of the wild-type IFITM5 with the FLAG-fused FKBP11 (FKBP11-FLAG) in the osteoblast cells in the absence and the presence of 2BP (denoted as “-” and “+”, respectively). Lanes 1 and 2 are the results for the control trials used to verify the existence of IFITM5 and FKBP11 before the immunoprecipitation, and Lanes 3 and 4 show the results after the immunoprecipitation. The experiment was repeated 3 times. B) Western blot for the co-immunoprecipitation of the wild-type and the cysteine-substituted mutants of IFITM5 with FKBP11-FLAG in the osteoblast cells. The band corresponding to FLAG peptide is not shown because of the smaller molecular-mass of FLAG peptide relative to FKBP11-FLAG. C) The control experiment of Figure 4-B used to verify that IFITM5 and FKBP11 were both present in the cell lysate before the immunoprecipitation. The experiment was repeated 2 times.

Previous studies have revealed that IFITM5 interacts with FKBP11 [19]. FKBP11 belongs to the FK506-binding protein family and has a transmembrane domain. The interaction between IFITM5 and FKBP11 is important for the immune activity because formation of the IFITM5-FKBP11-CD81-FPRP complex induces the expression of interferon-induced genes—namely, the *Bst2*, *Irgm*, *Ifit3*, *B2m*, and MHC class I antigen gene [28]. To investigate the effect of the S-palmitoylation on the interaction of IFITM5 with FKBP11, we carried out an immunoprecipitation assay. The osteoblast cells co-transfected by the plasmids encoding IFITM5-WT and FKBP11-FLAG were cultured in the absence and the presence of 2BP. Then, the extracted cell-lysate was incubated with anti-FLAG agarose gel. The gel was washed several times. Finally, the proteins were competitively eluted by the addition of FLAG peptide. If IFITM5 interacted with FKBP11, it was expected that IFITM5 would be obtained during this step and detected by immunoblotting. Figure 4-A shows the results of the western blot for the co-immunoprecipitation of IFITM5-WT with FKBP-FLAG. The band corresponding to FKBP11 appeared in all the lanes (upper panel). Lanes 1 and 2 are controls to ensure that IFITM5 and FKBP11 are both contained in the cell lysate before the immunoprecipitation. The controls also ensured that IFITM5 was S-palmitoylated in the absence of 2BP (see lane 1), whereas IFITM5 was not S-palmitoylated in the presence of 2BP (see lane 2). After the immunoprecipitation, a single band corresponding to the S-palmitoylated IFITM5 appeared in the absence of 2BP (see lane 3), indicating the interaction of the S-palmitoylated IFITM5 with FKBP11. However, in the presence of 2BP, no band corresponding to IFITM5 appeared (see lane 4), indicating that the two molecules do not interact with each other. These results suggest that the S-palmitoylation on IFITM5 contributes to the interaction with FKBP11.

### 4. Interaction of IFITM5 mutants with FKBP11

Next, we further investigated the relationship between the S-palmitoylation and the interaction with FKBP11 by using the IFITM5 mutants described above. The osteoblast cells co-transfected by the plasmids encoding IFITM5 mutants (C52A/C53A, C86A, and Cys-less) and FKBP11-FLAG were cultured. The immunoprecipitation assay was carried out in the same way as described above. Figure 4-B shows the results of the western blot for the co-immunoprecipitation of the wild-type and the IFITM5 mutants with FKBP11. Figure 4-C shows the results of the control experiment using the cell lysate before the immunoprecipitation. As described in the previous section *3-3*, the band corresponding to FKBP11 appeared in all the lanes (upper panels) because the immunoprecipitation was carried out using the anti-FLAG agarose gel. In the lower panel of Figure 4-B, single bands were observed for the IFITM5-WT and -C86A mutant (lanes 1 and 3) but not for the -C52A/C53A and Cys-less mutants (lanes 2 and 4). This result indicates that the wild-type and the C86A mutant interact with FKBP11, whereas the other two mutants do not. Interestingly, this tendency mirrored the trend for the S-palmitoylation profiles, which means that Cys52 and/or Cys53 in the TM1 domain of the IFITM5-WT and -C86A mutants is S-palmitoylated, whereas these residues are not S-palmitoylated in the C52A/C53A and Cys-less mutants (see Figures 2-D, 3-B and the lower panel of Figure 4-C). Because the S-palmitoylation contributes to the IFITM5-FKBP11 interaction, as described in the previous section *3-3* (also in Figure 4-A), the results of Figure 4-B suggest that the mutants which lost the S-palmitoylation site(s), Cys52 and/or Cys53, are not able to interact with FKBP11. In other words, the S-palmitoylation on these cysteines is necessary for the interaction of IFITM5 with FKBP11.

### 5. Role of S-palmitoylation on IFITM5 for bone nodule formation

**Figure 5:**
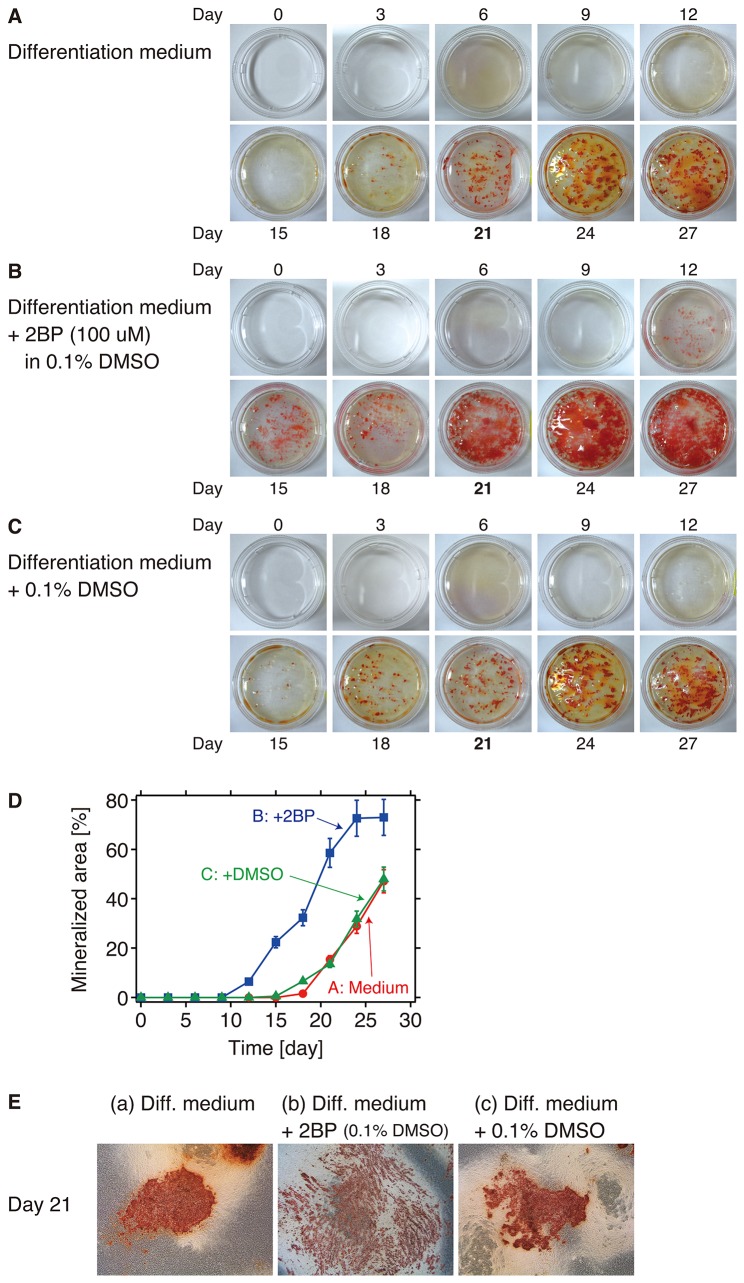
Bone nodule formation of osteoblast cells in the presence and absence of 2BP. A-C) Time-dependent bone nodule formation in the osteoblast cells A) in the absence of 2BP, B) in the presence of 2BP and 0.1% DMSO, and C) in the presence of 0.1% DMSO. The mineralized nodules are stained with Alizarin Red. D) The area occupied by the mineralized nodules (%) was plotted against the incubation time (day). Error bars indicate the standard error. E) Enlargement of the mineralized nodules on Day 21. In the presence of 2BP (panel b), the nodules were more diffuse (none were clustered) than in the other two conditions (panels a and c). On Day 0, the osteoblast cell differentiation was initiated. These experiments were carried out 3 times.

As described above in the *Introduction*, previous studies have revealed that IFITM5 also contributes to bone formation [18-21]. Therefore, we investigated the influence of S-palmitoylation on the bone nodule formation in osteoblast cells, in which native IFITM5 is expressed. Figure 5 shows the time-dependent nodule formation in the absence and the presence of 2BP (Figure 5-A and -B). Figure 5-C shows the results of the control trial to verify the effect of DMSO, which was used as the solvent for 2BP, on the nodule formation. The mineralized nodule was stained with Alizarin Red, which reacts with deposited calcium. In Figure 5-D, the area of the mineralized nodule was plotted against experimental time. In the absence of 2BP (Figure 5-A, -C, and -D), the mineralization was started 15 days after the initiation of the cell differentiation (Day 0). On the other hand, in the presence of 2BP (Figure 5-B and -D), the nodule was formed on Day 12. The halftime for the maximum mineralization in the presence of 2BP was estimated to be 7 days earlier than that in the absence of 2BP (Figure 5-D). In addition, differences in the form of the mineralized nodules were observed. Figure 5-E shows an enlarged view of each nodule on Day 21. The stained nodules were diffused in the presence of 2BP (panel b), whereas in the absence of 2BP the nodules formed a large cluster (panels a and c). Therefore, our observations in this study suggested that the S-palmitoylation affects the bone nodule formation in the osteoblast cells.

## Discussion

### 1. Comparison of IFITM5 with IFITM3 and IFITM2

In this study, we confirmed the S-palmitoylation on IFITM5 in the osteoblast cells, which was the same as that previously reported for IFITM3 and IFITM2. As reported previously, in IFITM3 and IFITM2, which share 85% sequence similarity (Figure 1-C), two cysteines in the TM1 domain (Cys71 and Cys72 for IFITM3, Cys70 and Cys71 for IFITM2) and one cysteine in the CP loop (Cys105 for IFITM3, Cys104 for IFITM2) are all S-palmitoylated in cells [10,24]. On the other hand, although IFITM5 shares 68% and 66% sequence similarity to IFITM3 and IFITM2, respectively, more than one cysteine in the TM1 domain (Cys52 or Cys53) and one cysteine in the CP loop (Cys86) are S-palmitoylated. Taking into account the high conservation of three cysteines in the IFITM proteins (Figures 1-A and 3-A), all the cysteines in IFITM5 may be involved in the S-palmitoylation just as in the case of IFITM3 and IFITM2 [10,24].

**Figure 6:**
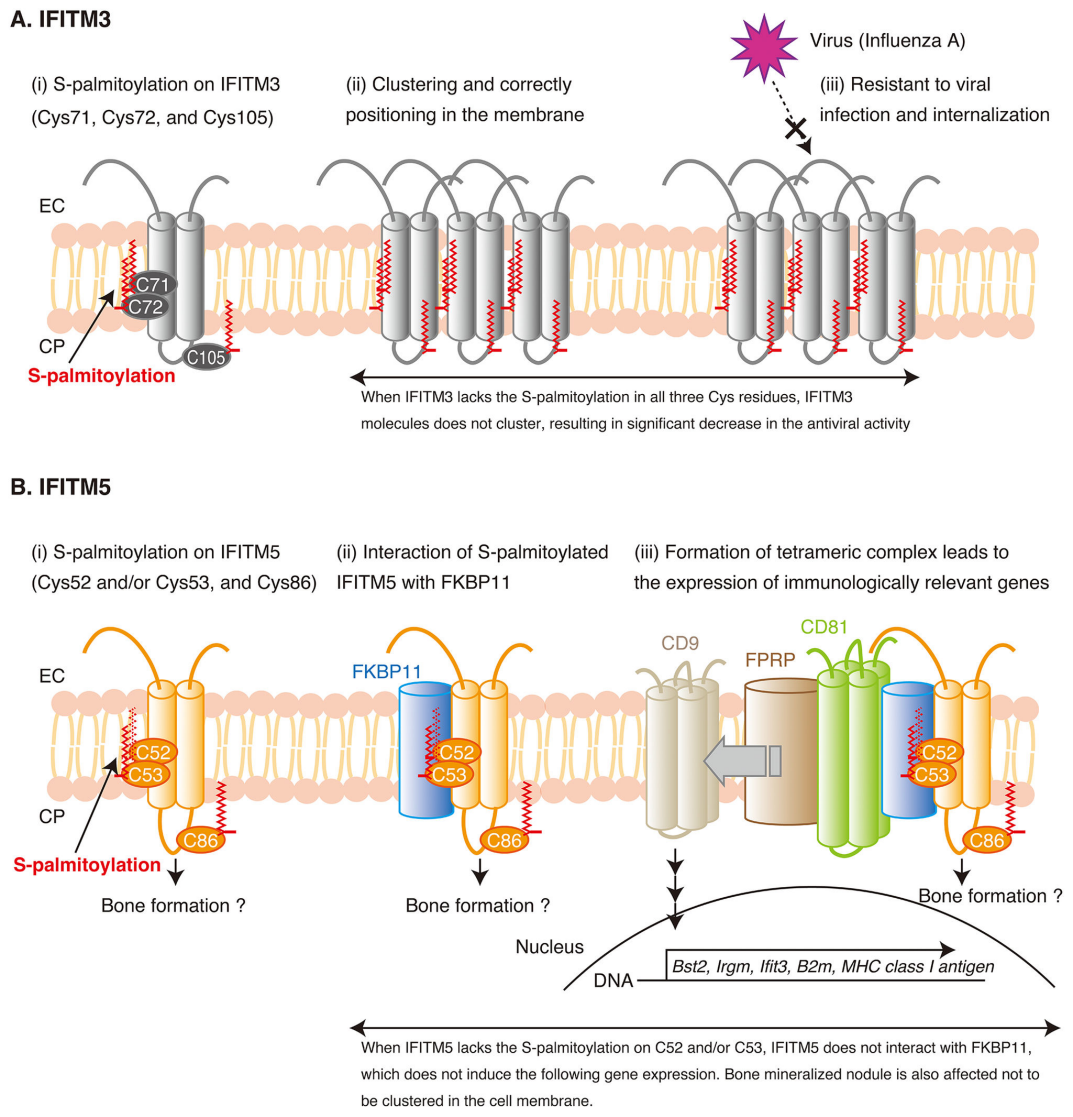
Possible functional mechanisms of IFITM3 and IFITM5 in cells. A) The functional mechanism of IFITM3 is summarized from previous studies. (i) IFITM3 is S-palmitoylated at Cys71, Cys72, and Cys105, (ii) which induces clustering and correct positioning in the membrane, (iii) resulting in the antiviral activity against influenza virus. B) The functional mechanism of IFITM5 is summarized by combining the results from the present and the previous studies. (i) Cys86, plus one or two other cysteine residues in IFITM5, i.e., Cys52 and/or Cys53, are S-palmitoylated (ii). The S-palmitoylation allows IFITM5 to interact with FKBP11 in the osteoblast cells (iii). The dissociation of CD9 from the FKBP11-CD81-FPRP/CD9 complex is induced by formation of the IFITM5-FKBP11-CD81-FPRP complex and leads to the immunologically relevant gene expression. IFITM5 also contributes to the bone formation, but it is unknown which states as described in (i)-(iii) are important for the bone formation at present.At present, no interactive protein has been identified in IFITM3 and IFITM2. On the other hand, IFITM5 interacts with the partner protein, FKBP11, and the S-palmitoylation clearly makes a significant contribution to the interaction. Therefore, IFITM5 forms a hetero-oligomer in the cell membrane for its physiological function.

The roles of the S-palmitoylation on IFITM3 have been studied intensively, and the S-palmitoylation has been shown to be crucial for the correct positioning in the membrane and the resistance to viral infection and internalization [10] (the roles are summarized in Figure 6-A and discussed in detail below). A recent study has revealed that the S-palmitoylation on IFITM2 is also important for the protein clustering in the membrane [24]. However, we do not know the role of the S-palmitoylation of IFITM5 for the clustering in the membrane at present because we have not yet succeeded in obtaining a proper antibody for immunohistochemistry, despite our allocating much time to the search and considering a considerable number of antibodies.

### 2. Role of S-palmitoylation in the IFITM5-FKBP11 interaction

Dr. Hanagata and co-workers previously reported that IFITM5 lacking the TM1 domain and the CP loop, which contain the relevant modification sites, lost the ability to interact with FKBP11 [19]. In the present study, we determined that the S-palmitoylation on Cys52 and/or Cys53 in the TM1 domain is necessary for the interaction. From these results, we speculate that Cys52 and Cys53 face toward the interaction surface with FKBP11, and therefore IFITM5 and FKBP11 interact with each other through the palmitic acid(s) attached to the cysteine(s) (summarized in Figure 6-B, discussed in detail later). Our investigation revealed that Cys86 is involved in the S-palmitoylation but does not contribute to the interaction with FKBP11. We speculate that some other residues in the CP loop located near the TM1 domain make some contribution to the interaction.

Previous investigations also revealed that IFITM5 expressed in the heterologous fibroblast NIH3T3 cells exhibited direct interactions with CD81, the B cell receptor-associated protein 31 (BCAP31), and the hydroxysteroid (17-beta) dehydrogenase 7 (HSD17b7). These three proteins bind to the IFITM5 without the S-palmitoylation (low molecular-mass form; see Figure 3-b in ref [19]. and Figure 1-B in ref [28].). In the fibroblast cells, the S-palmitoylation on IFITM5 is insufficient [19]. These interactions are not observed in the native osteoblast cells, and therefore are nonspecific. Taking these facts into consideration, we speculate that the S-palmitoylation on IFITM5 promotes the specific interaction with FKBP11 in the osteoblast cells.

### 3. Role of S-palmitoylation on IFITM5 for the osteoblast cell activity

The role played by the S-palmitoylation of IFITM5 in immune activity of the osteoblast cells will be discussed by combining the results from the present and the previous studies. A specific interaction between IFITM5 and FKBP11 should be necessary to form the IFITM5-FKBP11-CD81- FPRP complex. CD81, also known as TAPA-1, is a member of the tetraspanin membrane protein family and a component of the B-cell co-receptor complex which mediates the B-cell signaling for immune responses. When forming this complex, CD9, a partner protein with CD81, dissociates from the FKBP11-CD81-FPRP/CD9 complex and consequently induces the osteoblast-specific expression of the interferon-induced genes, *Bst2*, *Irgm*, *Ifit3*, *B2m*, and the MHC class I antigen gene [28]. If the S-palmitoylation-mediated specific interaction of IFITM5 with FKBP11 were lost, the IFITM5-FKBP11-CD81- FPRP complex would not be formed, and consequently the interferon-induced gene expression would be inhibited because CD9 would remain associated with the FKBP11-CD81-FPRP/CD9 complex. In this respect, we speculate that IFITM5 is involved in the immune system activity in the osteoblast cells and the interaction of the S-palmitoylated IFITM5 with FKBP11 regulates the immune activity.

In addition, it was suggested that the S-palmitoylation on IFITM5 contributes to the bone nodule formation, including morphology and time for mineralization, in the osteoblast cells (Figure 5). It is difficult to conclude at present that the lack of the S-palmitoylation on IFITM5 causes the diffusion of the bone nodules (panel b of Figure 5-E); we can say, however, that IFITM5 will probably not be S-palmitoylated in the cells in the presence of 2BP. While 2BP is commonly used as an inhibitor of palmitoylation, it also targets many metabolic enzymes [33,34]. Thus, it is also difficult to interpret the results of the long-term incubation of the osteoblast cells in the presence of 2BP. In any case, these are interesting and key observations in terms of clarifying the role played by the S-palmitoylation of IFITM5 in bone formation, and further studies are required.

### 4. Possible mechanism of the IFITM5-FKBP11 interaction and the cellular function

Figure 6 describes a possible mechanism of the interaction of IFITM5 with FKBP11 and the role of IFITM5 in the osteoblast cell function by means of a comparison with IFITM3. In the case of IFITM3, as shown in Figure 6-A, the following are observed. (i) The three cysteines are all S-palmitoylated (ii). The S-palmitoylation leads to the clustering and the correct positioning of IFITM3 molecules in the membrane (iii). The S-palmitoylation and the following clustering are crucial for the resistance to the influenza virus. When IFITM3 lacks the S-palmitoylation, the IFITM3 molecules do not cluster, which leads to the significant decrease in the antiviral activity.

On the other hand, Figure 6-B shows that the following observations are made in the case of IFITM5. (i) Cys86, plus one or two other cysteine residues in IFITM5, i.e., Cys52 and/or Cys53, are S-palmitoylated (ii). The S-palmitoylated IFITM5 is able to interact specifically with FKBP11. The interaction is presumed to be mediated by the palmitic acid(s) attached to the cysteine(s) facing toward the interaction surface on FKBP11. Cys86 is involved in the S-palmitoylation but not in the interaction of IFITM5 with FKBP11. At present, however, little is known about the role of the S-palmitoylation of IFITM5 for the localization in the membrane. When the S-palmitoylation affects the localization of IFITM5 as in the case of IFITM3 [10], the S-palmitoylated IFITM5 molecules should be localized in the membrane or the depalmitoylated molecules should be delocalized. The loss of the interaction between IFITM5 and FKBP11 could be due to a relocalization of the depalmitoylated IFITM5 that prevents its association with FKBP11 (iii). The S-palmitoylated IFITM5 interacts with the FKBP11-CD81-FPRP/CD9 complex through FKBP11, which induces the dissociation of CD9 from the complex and the expression of 5 immunologically relevant genes. Finally, IFITM5 forms the IFITM5-FKBP11-CD81-FPRP complex. It is unknown at present which of the three states (i)~(iii) illustrated in Figure 6-B is important for the bone mineralization of the osteoblast cells. The lack of the S-palmitoylation influences the interaction with FKBP11, which could account for the following complex formation and gene expression. In addition, the bone nodule formation is also affected. Note that the role of the S-palmitoylation has been involved in the bone formation [35]. It is indicated that the S-palmitoylation on IFITM5 plays roles not only for the regulation of the immune activity but also for the bone formation.

In conclusion, we have revealed the S-palmitoylation on IFITM5 and its role in the interaction with FKBP11. Not only the immune activity but also the bone mineralization in the osteoblast cells is affected by the S-palmitoylation. In general, the functional role of the S-palmitoylation is different for each protein [36]. For many proteins, the palmitoylation and depalmitoylation cycle is constitutive and regulated by enzymes. Based on the present results, it is difficult to address (i) whether the S-palmitoylation on IFITM5 is constitutive or regulated, or (ii) when and where IFITM5 is S-palmitoylated in the osteoblast cells. Further studies are required and are currently underway.
